# The influence of water potential in simulation: a catabolite activator protein case study

**DOI:** 10.1007/s00894-019-4095-3

**Published:** 2019-07-10

**Authors:** Steven Y. Liem, Paul L. A. Popelier

**Affiliations:** 1 Manchester Institute of Biotechnology (MIB), University of Manchester, 131 Princess Street, Manchester, M1 7DN Great Britain; 2 School of Chemistry, University of Manchester, Oxford Road, Manchester, M13 9PL Great Britain

**Keywords:** SPC, TIP3P, TIP4P, TIP5P, ff12SB, ff99SB, Force Field, Protein, Molecular dynamics

## Abstract

We present a rare comparison of structures of the same protein but generated by different potentials. We used four popular water potentials (SPC, TIP3P, TIP4P, TIP5P) in conjunction with the equally popular ff99SB. However, the ff12SB protein potential was used with TI3P only. Simulations (60 ns) were run on the catabolite activator protein (CAP), which is a textbook case of allosteric interaction. Overall, all potentials generated largely similar structures but failed to reproduce a crucial structural feature determined by NMR experiment. This example shows the need to develop next-generation potentials.

**Graphical abstract:**
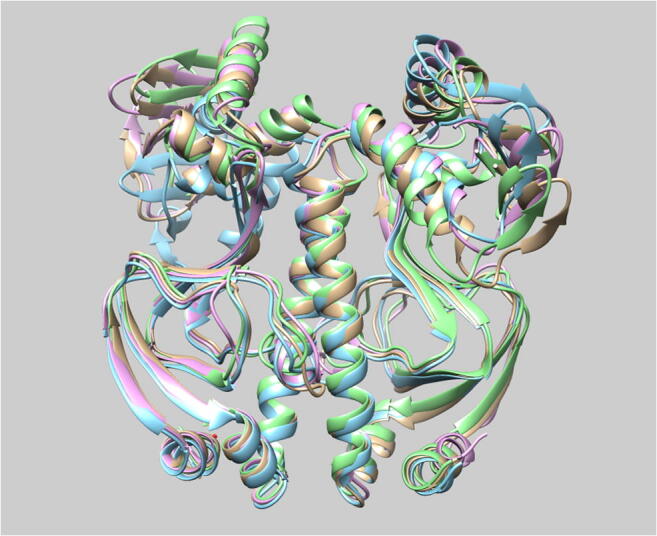
Catabolite activator protein

Catabolite activator protein

## Introduction

Molecular simulation has become the third way of doing science, next to theory and experiment. Powerful computers and algorithms provide a stream of information, independent from experiment, which sheds light on challenging biological systems and problems. Allostery is one such crucial and ubiquitous biochemical phenomenon, which consists of a triggering event at one site of a macromolecule leading to a corresponding effect at a distal site. For this phenomenon to be understood it is essential that experiment and simulation converge to the same view. This is why it is important that simulations connect with reality such that the virtual world they reveal becomes relevant to experiment. Of course, experiment also needs to make sure that it captures the correct underlying reality from the bare signals it detects. In fact, experiment tends to rely more and more on models and simulation in order to extract from its measured signals the desired structure, mechanism, and explanation of a variety of biochemical phenomena. For this symbiosis to be successful, one concern that needs to be met is the reliability of the energy potentials used in biomolecular simulation. The current work presents a relatively rare comparison between well-known potentials for water and for amino acids, and reports the effect of this variation on a case study involving allostery.

The nature of allostery is still not fully understood. Proteins in signal transduction pathways usually display an ultrasensitive cooperative response. Signal transduction pathways will fully reveal their secrets if we can accurately consider how each protein in a network processes information internally [1]. This is often accompanied by allosteric regulation [2] (“the second secret of life”). Allostery is the regulation of an enzyme by binding an effector molecule at the protein’s allosteric site [3], which is different from the protein’s active site. Evolution has made use of allostery: it plays an indispensable role in all processes in the living cell. In recent years interest in allosteric effectors acting as drug molecules has increased. However, the purpose of the current work is not to seek evidence for one model or the other but, as stated above, to find out how much varying the potentials affects the outcome of a simulation.

This case study features in the wider context of force field development, which is a research theme of our group. For many years we have advocated the use of multipolar electrostatics [4], which are known to be more accurate than point charges. This conclusion was reached by several labs (typically those that invest in developing next-generation force fields, such as AMOEBA [5], NEMO [6], SIBFA [7], XED [8], EFP [9], and DMACRYS [10] to name a few), as well as in our own work [11–14], for example, on liquid water using molecular dynamics (MD) simulations [15], or the systematic comparison between quantum topological [16, 17] multipole moments [18] and microhydration [19] of serine. The latter work carefully and systematically compares a host of geometrical features (angles and distances) against geometry-optimized ab initio structures, at static level, and radial and spatial distribution functions, at dynamic level. The four point-charge potentials that were compared returned very different results and can be ranked in terms of performance as follows, starting with the worst mean deviation in atomic positions compared with ab initio: TAFF [20] > OPLS-AA [21] > MMFF94x [22] > PFROSST [23]. Staying with (fixed) point-charge force fields, another striking example by Stock et al. [24] is that of a very small peptide casually called trialanine. Molecular dynamics simulations were run with six different force fields, namely two versions of AMBER [25] (parm94, parm96), two versions of GROMOS (43A1, 45A3), CHARMM [26] (1998), and OPLS [27] (all atom, 1996). Their conclusion was pessimistic: *“…it is not clear to what extent commonly used force fields are capable of correctly describing nonequilibrium dynamics such as the folding or unfolding of a peptide.”* Indeed, even the minor modification between AMBER’s “parm94” and “parm96” significantly changed the population ratio of the conformational states. Furthermore, the Ramachandran probability distribution plots from 20 ns MD simulations were *qualitatively* different between force fields. For example, OPLS could not resolve P_II_ and β, and AMBER “parm94” significantly populated the α-conformation.

More recently, in 2010, Verbaro et al. [28] looked at polyalanine peptides in solution and established that structural preferences in the unfolded state of peptides determined by MD still contradict experimental data. They conclude that “MD simulations suggesting more statistical coil-like distributions cannot be reconciled with spectroscopic data.” In 2015, Dean Smith et al. [29] examined the dynamics of an intrinsically disordered protein fragment of the amyloid β, the Aβ_21–30_ system, under seven commonly used force fields and three water models. Secondary structure measures and intrapeptide hydrogen-bonding differ significantly between force fields, with some force fields readily increasing helical content and the variety of intrapeptide hydrogen bonds. In the same year, another dramatic example [30] was published on the lack of reliability of intrinsically disordered proteins ensembles generated by eight all-atom empirical force fields when compared to primary small-angle X-ray scattering and NMR data. Ensembles obtained with different force fields exhibit marked differences in chain dimensions, hydrogen bonding, and secondary structure content. These differences are unexpectedly large: *changing the force field is found to have a stronger effect on secondary structure content than changing the entire peptide sequence!* This situation further motivates and justifies a truly novel approach started many years ago called FFLUX [31, 32], which is our in-house next-generation force field designed according to a completely different architecture to that of traditional point charge force fields. Meanwhile, to this day, systematic tests and comparisons [33, 34] continue to appear between popular force fields of standard architecture.

Molecular dynamics simulations carried out in parallel with different force fields are rare in the literature. Yet, this type of work is necessary to stimulate further development in the area of force fields. Here we conducted the first systematic investigation of the effect of water/protein potentials on the behavior of the catabolite activator protein (CAP) [35], in particular the apoenzyme. This protein is a classic system to study allostery with, especially because the mechanism by which allostery [36] occurs is still not settled although promising new proposals [37] are being made. The crystal structure [38] of Passner et al. is used as a starting point for our MD simulations. Other than comparisons between the simulations carried out by different potentials, we will also refer to the NMR study [39] by Popovych et al., henceforth referred to as *Paper A*.

It should be noted that point-charge-based biomolecular force fields are normally only meant to be used with the water model against which they were parameterized. However, we considered water as a special case because much effort has been devoted in formulating an accurate potential that is capable of reproducing many experimental results. It is of interest to see the effects of such potentials in conjunction with a biomolecular force field, which is the subject of the current article.

## Materials and methods

### An allostery case study

**Fig. 1:**
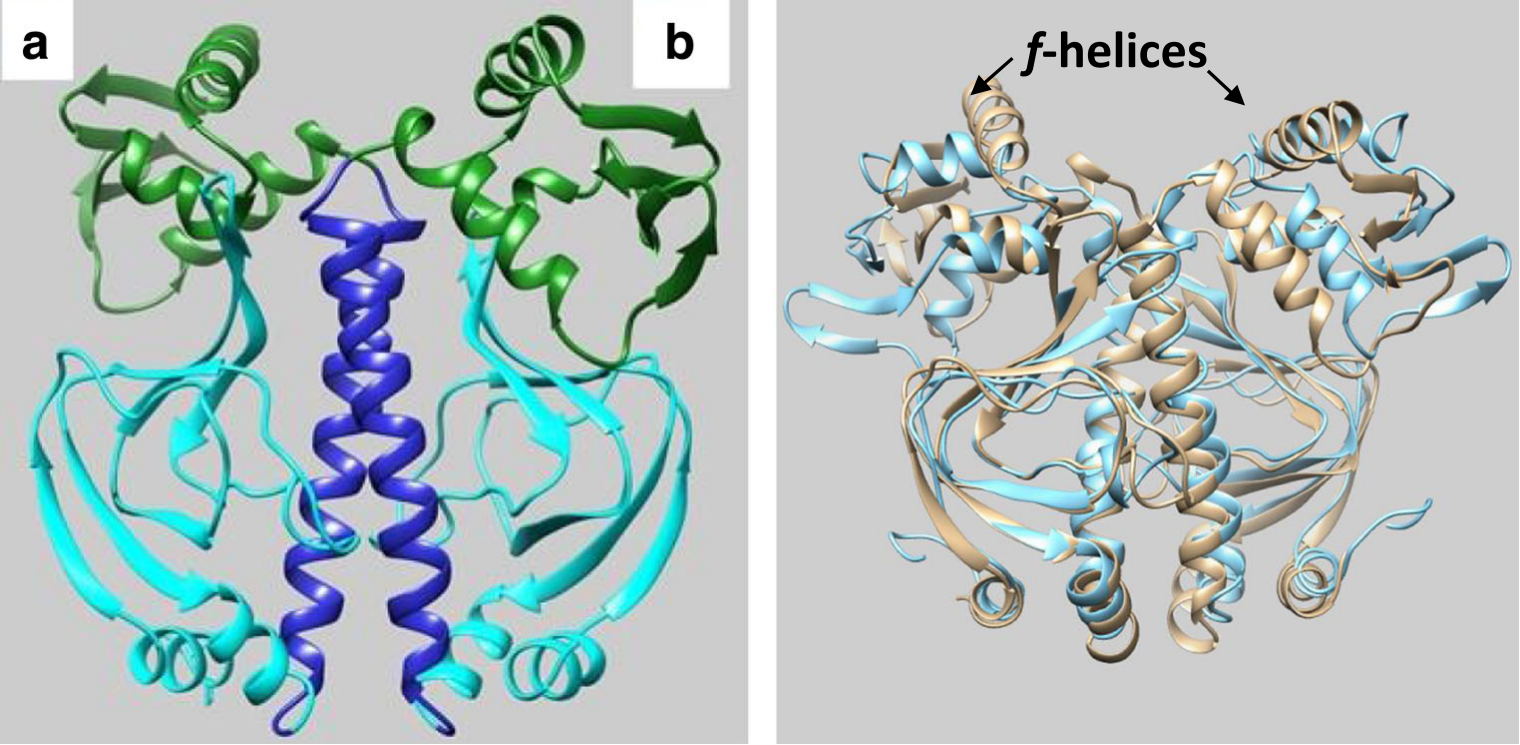
In the left panel, the ribbon representation of the two ligand-binding subunits (cyan), two DNA-binding subunits (green), and the central helices (blue) of *apo*-CAP in 1G6N. In this work, we refer to the left and right subunits as “A” and “B”, respectively (e.g., ligand-A). The right panel shows the comparison of 1G6N (gold) and the first fragment of 2WC2 (light cyan, from Paper A)

For our current case study, we chose a fascinating example of an experimentally, but also computationally, investigated [3, 40–42] allosteric protein called CAP, which is involved in the transcription of DNA. It is a 47 kDa homodimer, and each of the two identical constituent monomers contains two subunits and a hinge region connecting those subunits: one is a specific binding site for DNA, the C-terminus, and the other is a specific binding site for a ligand such as cAMP (cyclic adenosine monophospate), the N-terminus. The Protein Data Bank [43] structure [38] 1G6N for CAP was used as the starting point for our MD simulations. This structure was also used in the MD simulations [40] of Li et al. The left panel of Fig. 1 shows a ribbon representation of the *apo* structure of this system, which consists of two subunits called *A* and *B*. In turn, each subunit consists of a DNA-binding subunit (top) and a ligand-binding subunit (bottom). Hence, there are four possible regions: DNA-A, DNA-B, ligand-A, and ligand-B. Finally, there are two central helices. Figure 1 (right panel) show the comparison between our starting structure (1G6N) and that proposed in Paper A (2WC2). It is clear that the two structures are reasonably similar. However, in both DNA binding subunits (right panel, top), the helix of Paper A (called *f-helix*) is almost perpendicular to the corresponding helix in our starting structure.

### Computational details

In this study we contrasted the dynamic behavior and conformation of the solvated CAP system obtained by NMR spectroscopy as reported in Paper A, with our own results obtained from four different water potentials: SPC [44], TIP3P [45], TIP4P [46], and TIP5P [47]. For TIP3P water, we also examined the differences due to the use of ff99SB and ff12SB protein potentials for the CAP molecule.

The preparation of the initial configuration involves the removal of water and the ligand molecules in the structure by using the program *Chimera* [48]. Missing hydrogen atoms were added subsequently and atomic charges of nonstandard residues were adjusted by using the AM1-BCC [49, 50] methodology [51]. The last step of the preparation is to solvate the *apo*-CAP protein with a layer of water molecules of the desired type (i.e., SPC or TIP*n*P, where *n* = 3, 4 or 5) and a thickness that is deemed sufficient to make the protein “experience” that it is in bulk water. During our initial investigation, we found that a system with a 12 Å thick water layer suffices for our purpose. Indeed, a thicker layer will not produce notably different results and only use considerably more computing time. Table 1 summarizes the details of the systems. The solvated system is then minimized in the NVT ensemble (with periodic boundary conditions) using the program *sander* of the AMBER package. Subsequently, the minimized system was allowed to equilibrate at 300 K for 2 ns before production runs (total of 60 ns) in the NpT ensemble were carried out.

**Table 1:** Details of the systems under study and their simulations

Potential^a^	Number of water molecules	Box dimension^b^ (Å × Å × Å)	Computation speed (ns/day)
SPC	17,802	75.2 × 91.3 × 85.9	20.9
TIP3P	17,802	75.5 × 91.7 × 86.3	20.9
TIP4P	17,682	75.2 × 91.4 × 85.7	13.9
TIP5P	17,715	74.9 × 92.0 × 86.4	10.6
TIP3P (ff12SB)	17,802	75.5 × 91.7 × 86.3	20.8

^a^Unless stated otherwise the protein potential is ff99SB

^b^The average dimension of the simulation box for production runs

All MD simulation runs were carried out using the CUDA enabled “pmemd” program of AMBER 12. We did not impose rotational restraints in our simulations as the box is quasi-cubic (Table 1). The smallest dimension is still larger than the long axis of the CAP molecule. A potential cutoff of 10 Å was adopted for Lennard-Jones interactions and the SHAKE [52] algorithm was employed to constrain all bonds that involve hydrogen atoms, which enables a time step size of 2 fs to integrate the equations of motion. The Berendsen loose-coupling algorithms [53] for temperature and pressure were used to maintain the temperature and pressure of the system at 300 K and 1 atm, respectively. The relaxation time for pressure and temperature were set to be 1 ps. Snapshots of the system were stored every 20 ps to facilitate post-simulation analysis.

### Analysis of simulations

As an initial analysis, we calculated an averaged structure of the CAP molecule generated from the simulations. The resulting averaged structures are visually inspected by using the program *Chimera*. We also monitored the root-mean-square-deviation (RMSD) of the two ligand-binding subunits and the two DNA-binding subunits in order to delineate any significant variation in their dynamic behavior that could be caused by the use of different potentials. The RMSD uses the starting configuration as a reference, and represents the average over the positions of all relevant atoms (i.e., those atoms that appear in the part of the system being monitored). We also calculated the standard deviation of the RMSD values for each of the subunits.

Finally, we calculated the diffusion coefficient, *D*, of the water molecules from the mean square displacement of the water molecules from the trajectory of the MD simulation. The Einstein relation links *D* to the mean square displacement:

1 $$ D=\underset{\mathrm{t}\to \infty }{\lim}\frac{1}{6{N}_wt}\left\langle {\sum}_{i=1}^{N_w}{\left[{\boldsymbol{r}}_i(t)-{\boldsymbol{r}}_i(0)\right]}^2\right\rangle $$

where *N*_*w*_ is the number of water molecules in the system, *t* is a certain point in time during the simulation, and ***r***_*i*_ is the position of the *i*th water molecule. The mean square displacement can be calculated from the trajectory of a simulation by using the built-in command “diffusion” of “ptraj”. The value of *D* can be evaluated from the best fitted slope of a plot of mean square displacement against time. Note that the nonlinear part in not noticeable in the graphs used to determine *D*, which we believe is due to long time spacing (10 ps) between the data points.

## Results and discussion

The averaged densities and energetics of the systems (see Table 2) are reasonably similar indicating that all potentials produce realistic system densities. This result should not be taken for granted because it is quite remarkable that the addition of CAP to an originally pure liquid water system largely preserves the latter’s density. Secondly, the respective variation in potential energy and density is only 5% and 1% for the systems that we used in this study. This ensures that our comparison is meaningful because the systems under study are in similar states. The computed diffusion coefficients using the first segment of the production run (length of 20 ns) vary by 54% (range versus mean). This considerable variation between these standard potentials is known to exist for pure liquid water. The presence of the protein preserves the relative ordering of the values of the diffusion coefficients, and enhances them by 1% up to 16%. The only exception is for TIP3P when used in conjunction with ff12SB, where the diffusion coefficient is slightly reduced. In fact, the presence of the protein has the smallest effect when using TIP3P.

**Table 2:** Comparison of averaged system density, total potential energy, and diffusion coefficient of water molecules obtained by any of the five potentials investigated

Potential^a^	Density (g cm^−3^)	Potential energy (kcal mol^−1^)	Diffusion coefficient^b^ (10^−5^ cm^2^ s^−1^)
SPC	1.029	−191.2	4.44 ± 0.06 (3.85, +16%)
TIP3P	1.017	−181.7	5.21 ± 0.05 (5.19, +0.4%)
TIP4P	1.026	−186.6	3.43 ± 0.04 (3.29, +4%)
TIP5P	1.017	−181.5	2.99 ± 0.15 (2.62, +14%)
TIP3P (ff12SB)	1.017	−182.8	5.23 ± 0.06 (5.19, −0.8%)

^a^Unless stated otherwise the protein potential is ff99SB

^b^The values in brackets correspond to values for pure liquid water calculated using the corresponding potential (extracted from http://www1.lsbu.ac.uk/water/water_models.html). For each potential the difference between the pure liquid and our calculated value is given as a percentage difference

The parallel behavior of the potential in going from pure water to CAP in water is perhaps not surprising because our systems can be considered as similar to pure water systems. This similarity is due to the fact that only water molecules adjacent to the CAP molecule will behave differently, while those waters in the bulk will behave similarly to pure liquid water. Indeed, the diffusion coefficient is dominated by the bulk water. However, we note that the value of the diffusion coefficient for the SPC and TIP5P system is more than 10% larger than that for the pure system. We believe that the higher value could be due to the presence of the *apo*-CAP molecule in the system, which curiously enhances the mobility of the water molecule in the SPC and TIP5P systems.

**Fig. 2:**
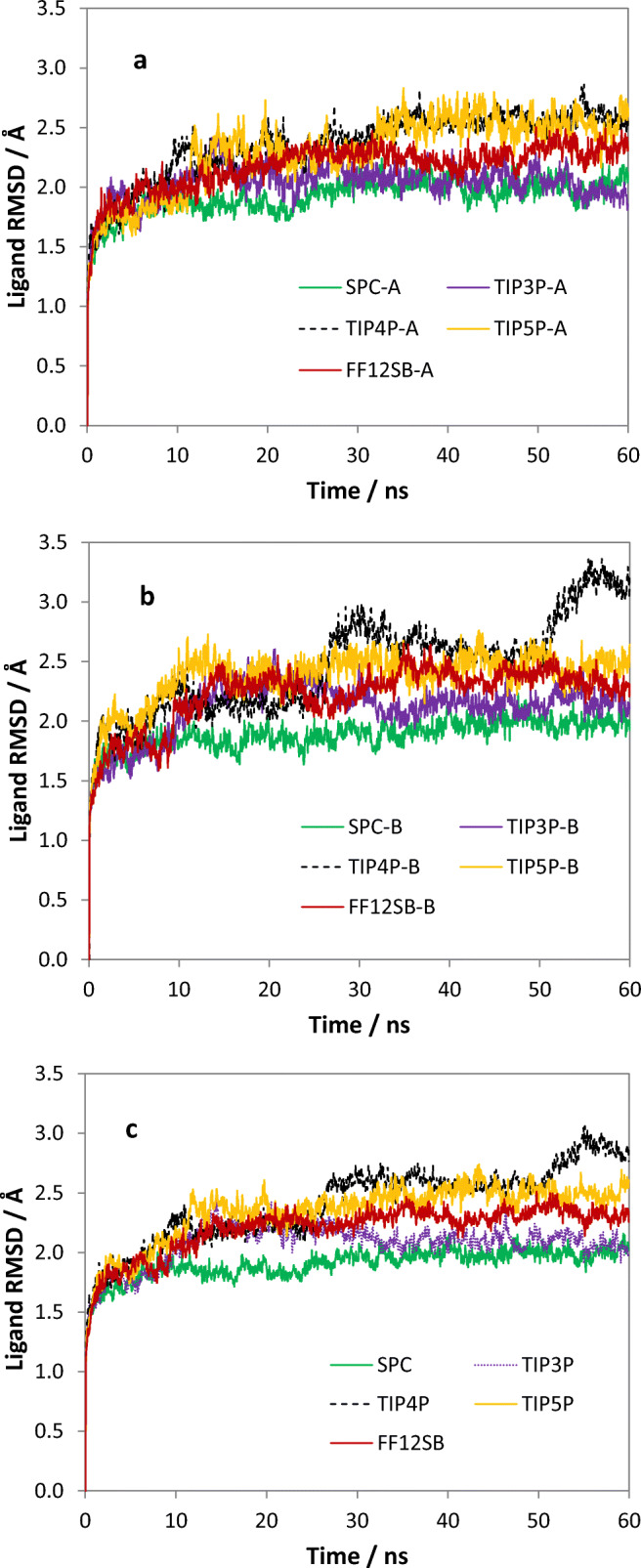
Variation of the RMSD values over the full 60 ns simulation for the (a) ligand-binding subunits in *A*, (b) in *B*, and (c) the mean of *A* and *B*

Figure 2 shows the results for the RMSDs of the two ligand-binding subunits. Apart from the TIP4P potential, the behavior of the *A* and *B* subunits are similar between all potentials. The general trend is that the RMSD values gradually increase after the initial sharp rise and reach a plateau value after about 20 to 25 ns. However, the *B* subunit of the TIP4P system exhibits large change during the latter part of the production run (around 25 and 50 ns). Nevertheless, the structure of the subunit remains intact upon visual inspection of this part of the trajectory. In addition, our result indicates that the *B* subunit does not have a strong influence on the *A* subunit because its RMSD does not exhibit any unusual fluctuation in the same time period. A visual inspection of the trajectories (in plots not shown in the paper) demonstrates that *each* subunit evolves according to its environment. Each subunit can change shape substantially from its initial configuration. Figure 2c shows the RMSD values averaged between ligand *A* and *B*. This new measure fluctuates less with progressing time, due to compensatory effects between ligand *A* and *B*. Given that the structures of these two subunits start off being the same, their individual progression provides twice as many data as for one subunit. Thus, this averaged RMSD value offers the advantage of giving a clearer impression of variation in RMSD between the five different simulations. Table 3 shows that this RMSD value (averaged of the two ligand-binding subunits) is ordered as follows: TIP4P > TIP5P > TIP3P/ff12SB > TIP3P > SPC. This trend is also followed by the values of the standard deviations.

**Table 3:** Mean RMSD values with respective standard deviations of the ligand-binding and DNA-binding subunits

Potential^a^	Mean RMSD	
	Ligand	DNA
SPC	1.91 ± 0.14	2.24 ± 0.27
TIP3P	2.07 ± 0.19	2.48 ± 0.25
TIP4P	2.41 ± 0.34	2.76 ± 0.41
TIP5P	2.34 ± 0.27	2.33 ± 0.25
TIP3P (ff12SB)	2.20 ± 0.22	2.23 ± 0.24

^a^Unless stated otherwise the protein potential is ff99SB

**Fig. 3:**
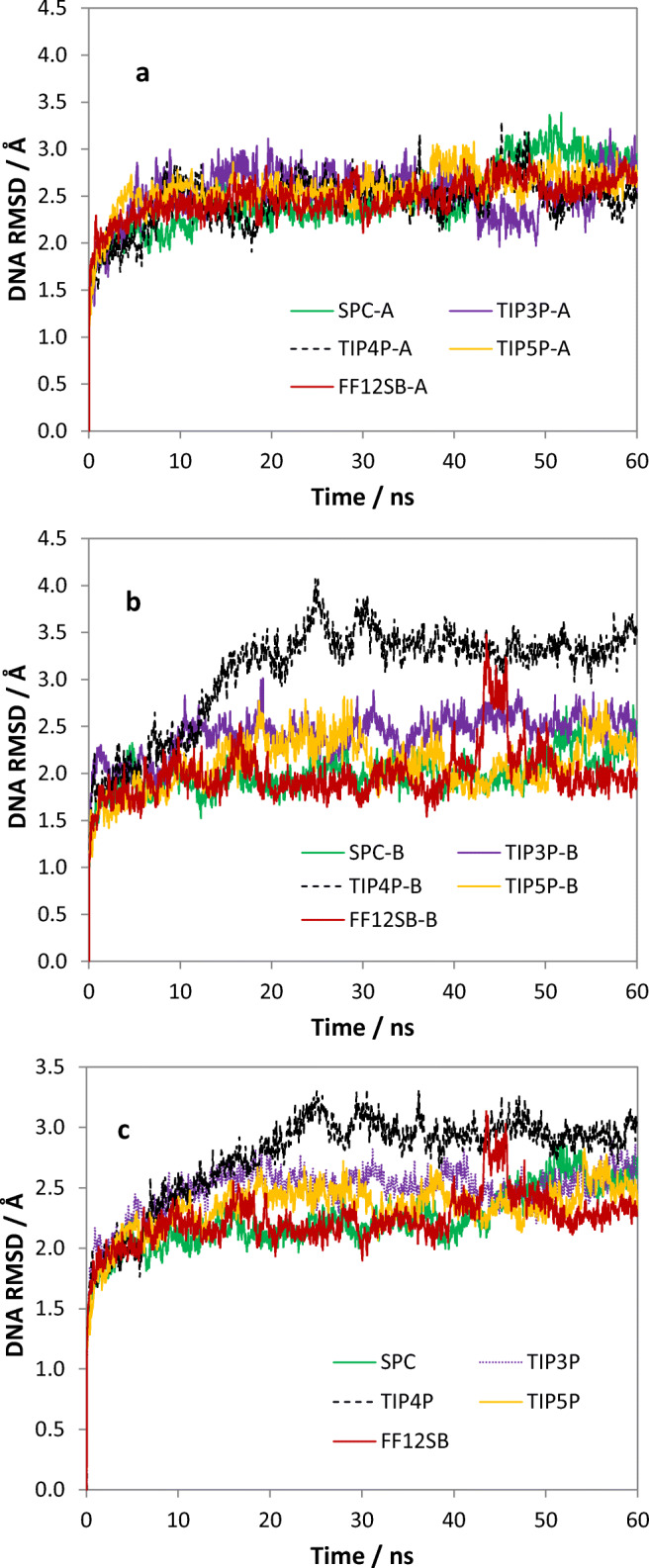
Variation of the RMSD values over the full 60 ns simulation for the (a) DNA-binding subunits in *A*, (b) in *B*, and (c) the mean of *A* and *B*

The results for the RMSDs for the DNA-binding subunits are shown in Fig. 3, and the general trend of the RMSDs is similar to those of the ligand-binding subunits. The *B* subunit of the TIP4P system attains the highest plateau value and exhibits some quite significant change between 20 and 30 ns. The *B* subunit of the ff12SB system also displays a notable but short lived increase near the end of the simulation (around 45 ns). However, the large variation in RMSD of the *B* subunit seems to have no obvious impact on the A subunit in either systems. Figure 3c shows the RMSD values averaged between DNA subunits *A* and *B*. The significant increase for TIP4P is now reduced because of averaging effects between the two subunits. Table 3 shows that the mean RMSD value (of the DNA-binding subunits) is ordered as follows: TIP4P > TIP3P > TIP5P > SPC ≈ TIP3P/ff12SB, while the order for the standard deviations is TIP4P > SPC > TIP5P ≈ TIP3P > TIP3P/ff12SB.

Table 3 shows that the TIP4P system has the highest mean value for the RMSD and the standard deviation for both ligand- and DNA-binding subunits. This probably means that the subunits of the TIP4P system are more flexible than those in the other four systems. The SPC system seems to have the least flexibility considering three out of the four calculated values are near the bottom. We combine this fact with the fact that SPC has the highest diffusion coefficient (see Table 2). Thus, the mobility of the water molecules does not have a large impact on the dynamic character of the subunits. Finally, the TIP3P/ff99SB and TIP3P/ff12SB potentials differ little based on their standard deviations but have substantially different mean RMSD values for both DNA- and ligand-binding subunits.

**Fig. 4:**
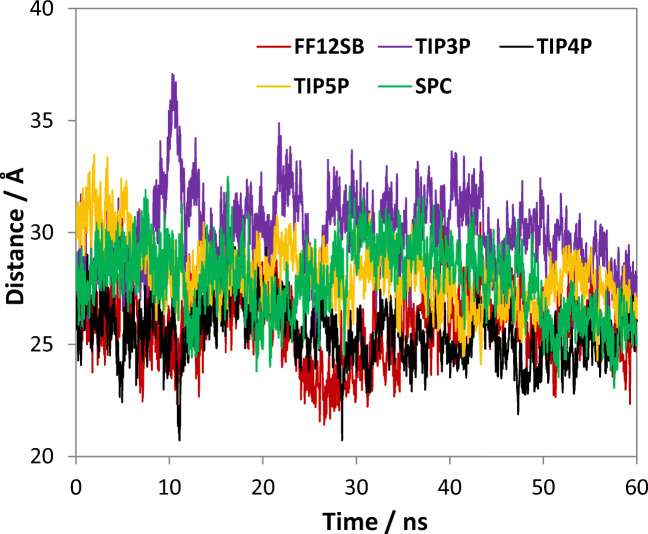
Variation of the distance between the center-of-mass of the two f-helices in the DNA binding subunits over the full 60 ns simulation

Apart from monitoring the variation of various RMSD values, we have also examined the fluctuations in distance between the two f-helices in the DNA binding subunits (Fig. 4). This distance is determined to be 41 Å for structures in 2WC2, and 34 Å when the CAP molecule binds to DNA (Paper A). It should be noted that the orientation of the f-helices also undergoes a 60° rotation when bound to DNA. For our own study, the f-helices did not undergo such a rotation because the orientation of the f-helices in the 1G6N structure is almost perpendicular to that in 2WC2. Our results show that the distance does vary in the course of the simulation but that it does not have any correlation with the variation observed in the RMSD plots. The average inter-helix distance obtained for all our water potentials ranges between 26 Å and 30 Å, which is significantly shorter than the range determined in Paper A. This is perhaps not surprising because the influence of the DNA molecule is absent in our system.

**Fig. 5:**
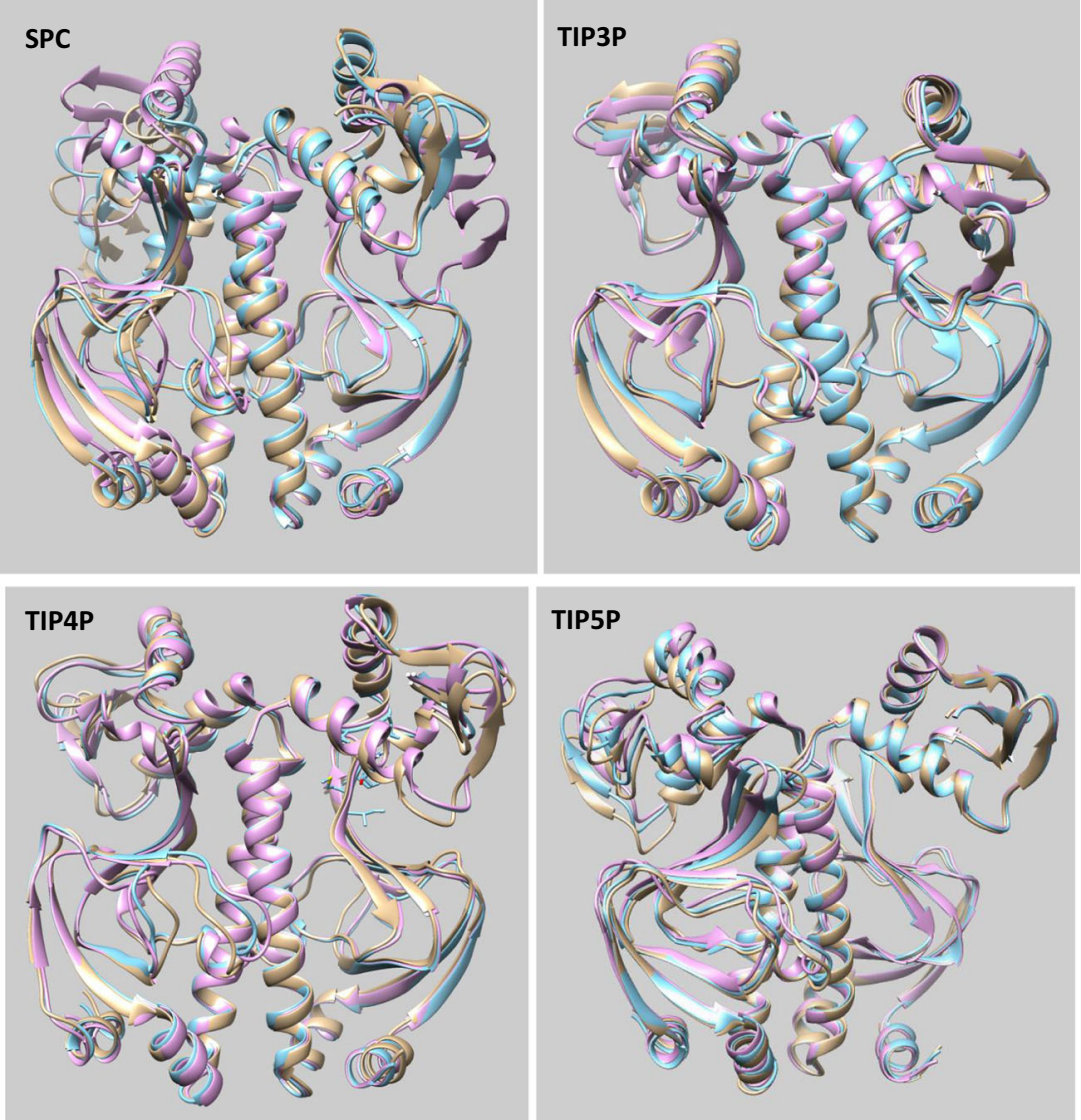
Comparison of averaged structures from consecutive runs using four different water potentials (SPC, TIP3P, TIP4P, and TIP5P). The three colors used correspond to three different simulation intervals: 0–20 ns (gold), 20–40 ns (cyan), and 40–60 ns (magenta). DNA- and ligand-binding subunits are at the top and the bottom, respectively

A more direct examination of the differences between the various systems was carried out by comparing the averaged structures evaluated from the production run. For each production run of 60 ns, we evaluated three averaged structures, each of which corresponds to 20 ns of consecutive simulation runs. Using the program *Chimera* (See Section 2.3), Fig. 5 shows the large similarity, observed for each water potential used with ff99SB, between the averaged structures appearing in the three time windows of 20 ns each. Indeed, many parts of the system overlap to a great extent between the time windows. Unlike the SPC system, the ligand- and DNA-binding subunits of the averaged structures in the TIP*n*P systems show very limited variation and overlap very well with each other. For the SPC system, the ligand-binding subunits in the averaged structures coincide quite well but the left DNA-binding subunit does show large deviations. Figure 5 also shows that the orientation of the f-helices in the DNA-binding domain is largely similar in the averaged structures corresponding to each of the three time windows. However, visual inspection of the snapshots occurring in the simulation trajectory (not shown) demonstrate some deviation in the *f* helices from their averaged structure.

**Fig. 6:**
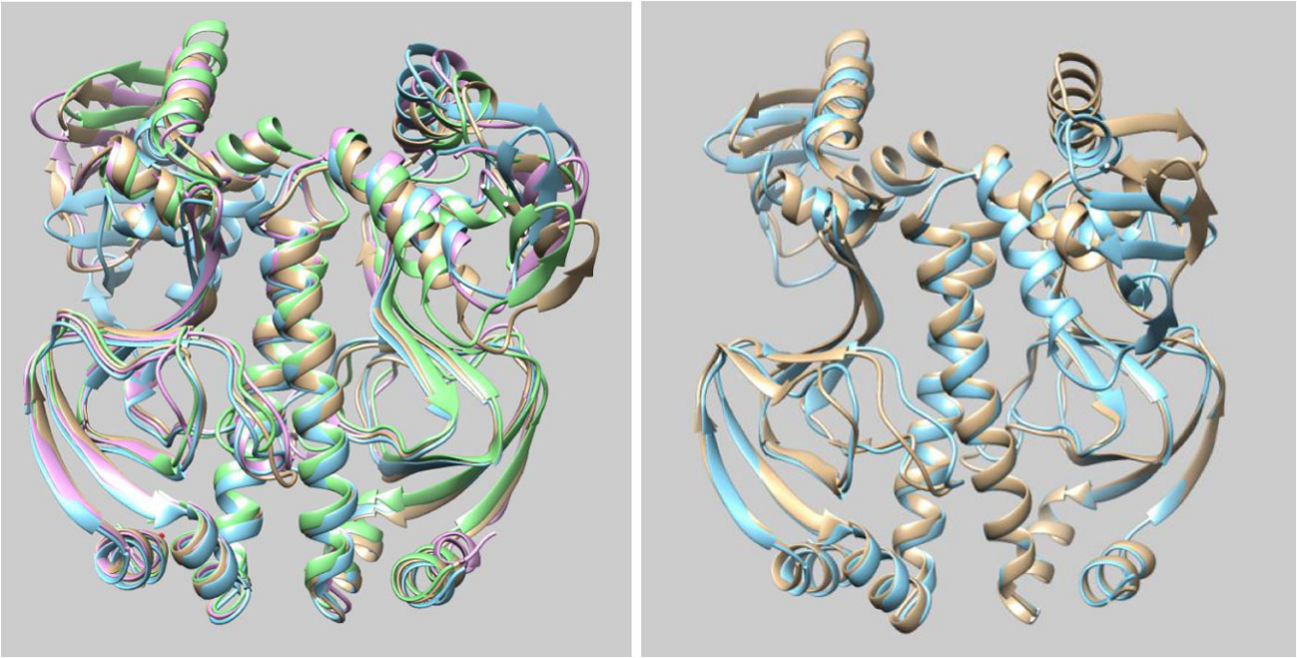
Comparison of structures averaged over 60 ns. Left panel: SPC (gold), TIP3P (cyan), TIP4P (magenta), and TIP5P (green) water potentials. Right panel: ff12SB (gold) and ff99SB (cyan) potentials for CAP. DNA- and ligand-binding subunits are at the top and the bottom, respectively

Figure 6 shows the comparison of overall averaged structure for each system using the entire 60 ns trajectory to produce a single averaged structure. The left panel compares four water potentials used alongside ff99SB, and the right panel only the TIP3P water potential used alongside ff99SB and ff12SB. Figure 6 (left panel) demonstrates the surprising conformational similarity in the ligand-binding subunits of the different water potentials because again those parts of the CAP strongly overlap. However, the DNA-subunits do show more variation between the four water potentials but still possess some degree of similarity, which is illustrated by the alignment of the helices in the subunit. The top two helices play a vital role in the binding of DNAs [see Paper A]. The same degrees of similarity, for both DNA- and ligand-binding subunits, were noted using both ff99SB and ff12SB for CAP (right panel of Fig. 6).

**Fig. 7:**
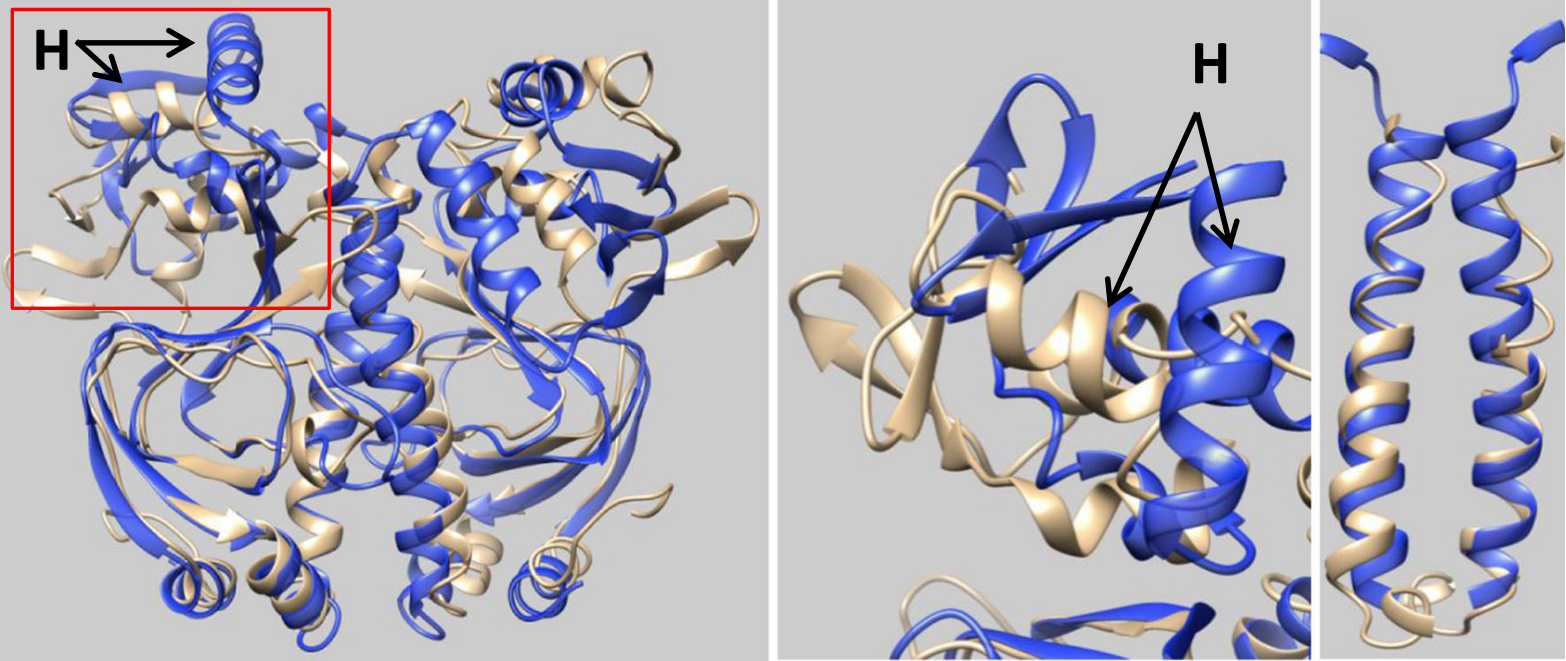
Comparison between the averaged structure obtained with TIP3P/ff99SB (blue) and that of Paper A (gold, first structure of the suggested conformations). The middle panel corresponds to the region enclosed by the red rectangle of the left panel but now viewed from the top. The label “H” indicates the top helices that are believed to play an important role in binding to DNA. The right panel focuses on the central helices where the top part of the helices (gold) are clearly uncoiled

Now we compare the structure suggested in Paper A with those obtained in this work. As the overall conformations of the CAP molecules are largely insensitive to the potential used, we picked the averaged structure from the TIP3P system for this comparison. The left panel of Fig. 7 gives an overall view of how the two structures compare to each other. The ligand-binding subunits are reasonably comparable, while the DNA subunits display large differences. The right panel of Fig. 7 presents an enlarged view of the left DNA subunit, and it clearly shows that the top helices (labelled “H” in the picture) have completely different orientations. In addition, there are also some notable variations in the structure of the two central vertical helices. Paper A observed partial unwinding of the coiled coil (top part of the central helices) for *apo*-CAP. This can be clearly seen in the right panel of Fig. 7, where the unwound coiled coil is represented as a tube. It is quite obvious that the corresponding section in the TIP3P system still retains its helical structure (represented as a ribbon). These two differences have also been observed for systems using other water potentials as well as structures obtained using the ff12SB potential (see Fig. 6, right panel).

On the basis of NMR relaxation data, Paper A indicated that the unwound coiled coil underwent substantial motions on a pico-to-nanosecond time scale, which the authors associated with enhanced flexibility. They also concluded that the unwinding of the coiled coil ultimately enables the DNA subunits to adopt a rather different orientation. It is important to point out that all our simulations occurred for a single CAP molecule solvated by different water models. Our results show that this part of the helix remains stable and the orientation of the f-helices remains largely unchanged for the entire duration of the simulations (60 ns).

## Conclusions

In this study, we compared and contrasted the behavior and conformation of a single solvated catabolite activator protein (CAP) using four different water potentials (SPC, TIP3P, TIP4P, and TIP5P) with the ff99SB protein potential, while TIP3P was also used with the ff12SB protein potential. The simulated systems have comparable densities and total potential energies. In addition, the diffusion coefficients of the water molecules are largely similar to those of the corresponding pure liquid water. However, for SPC and TIP5P, diffusion coefficients calculated from our simulations using the specific water potential are higher than that of pure liquid water by more than 10%. This enhancement is an unexpected effect of using the SPC and TIP5P water potential in conjunction with ff99SB potential.

We used the RMSD of the ligand- and DNA-binding subunits to examine the dynamic behavior of the systems. The results show that, apart from the TIP4P system, the RMSDs of the subunits are quite similar. The TIP4P system has a higher RMSD value and also exhibits larger fluctuations for both ligand- and DNA-binding subunits. Nevertheless, the comparison of the averaged structures shows that overall conformations are very similar. This is especially the case for the ligand-binding subunits where the structures largely overlap each other. However, the DNA subunits overlap less but the helices still exhibit some degree of alignment.

The most important observation is that the orientation of the f-helices in the DNA subunit is largely unchanged. In addition, we do not see the partial unwinding of the top part of the central vertical helix in any of our simulations. We believe that this different behavior is simply due a different simulation context, both by differences in starting geometry of the system and its molecular make-up (presence or absence of DNA).
